# COVID-19 Vaccination and Public Health: Addressing Global, Regional, and Within-Country Inequalities

**DOI:** 10.3390/vaccines12080885

**Published:** 2024-08-04

**Authors:** Omar Enzo Santangelo, Sandro Provenzano, Giuseppe Di Martino, Pietro Ferrara

**Affiliations:** 1 Regional Health Care and Social Agency of Lodi, ASST Lodi, 26900 Lodi, Italy; 2 School of Medicine and Surgery, University of Milan, 20122 Milan, Italy; 3 Local Health Unit of Trapani, ASP Trapani, 91100 Trapani, Italy; 4 Department of Medicine and Ageing Sciences, “G. d’Annunzio” University of Chieti-Pescara, 66100 Chieti, Italy; 5 Unit of Hygiene, Epidemiology and Public Health, Local Health Authority of Pescara, 65100 Pescara, Italy; 6 Center for Public Health Research, University of Milan–Bicocca, 20900 Monza, Italy; 7 Laboratory of Public Health, IRCCS Istituto Auxologico Italiano, 20149 Milan, Italy

**Keywords:** COVID-19 vaccine, health inequalities, population impact of COVID-19 vaccination, vaccine access

## Abstract

The COVID-19 pandemic, with over 775 million cases and 7 million deaths by May 2024, has drastically impacted global public health and exacerbated existing healthcare inequalities. The swift development and distribution of COVID-19 vaccines have been critical in combating the virus, yet disparities in access to and administration of the vaccine have highlighted deep-seated inequities at global, regional, and national levels. Wealthier nations have benefited from early access to vaccines, while low- and middle-income countries (LMICs) have faced persistent shortages. Initiatives such as COVAX aimed to address these disparities, but challenges persist. Socioeconomic factors, education, ethnic identity, and the healthcare infrastructure play crucial roles in vaccine equity. For example, lower-income individuals often face barriers such as poor access to healthcare, misinformation, and logistical challenges, particularly in rural areas. Addressing these inequities requires a multifaceted approach, integrating national policies with local strategies to enhance vaccines’ accessibility, counter misinformation, and ensure equitable distribution. Collaborative efforts at all levels are essential to promote vaccine equity and effectively control the pandemic, ensuring that all populations have fair access to life-saving vaccines. This review explores these complex issues, offering insights into the barriers and facilitators of vaccine equity and providing recommendations to promote more equitable and effective vaccination programs. With a focus on the different levels at which vaccination policies are planned and implemented, the text provides guidelines to steer vaccination strategies, emphasizing the role of international cooperation and local policy frameworks as keys to achieving equitable vaccination coverage.

## 1. Introduction to COVID-19 Vaccination Inequity

With over 775 million cases and 7 million deaths by the end of May 2024, the coronavirus disease 2019 (COVID-19) pandemic has created previously unheard-of difficulties for global public health. Indeed, it has had a profound effect on all facets of society, from economies to healthcare systems, and it has made major gaps in access to and outcomes of healthcare worse in a variety of contexts [1,2,3,4].

A key development in the fight against COVID-19 was the swift development and introduction of the vaccines, which served as the foundation for public health initiatives aimed at containing and lessening the pandemic’s catastrophic effects. A number of vaccination candidates have expeditiously been authorized for emergency use, utilizing advancements in biotechnology such messenger RNA (mRNA) vaccine platforms [5,6].

Since the beginning of the global vaccination campaigns and as they progressed, the distribution and administration of COVID-19 vaccines and vaccination strategies have, however, highlighted significant disparities at the global, regional, and within-country levels, underscoring the deep-seated inequalities in healthcare across populations and groups [7,8,9].

Wealthier nations have largely benefited from early and extensive access to COVID-19 vaccines for the initial vaccination campaigns of 2021 and for booster doses, often securing vast supplies through pre-purchase agreements, while many low- and middle-income countries (LMICs) have struggled to obtain sufficient doses for their populations [10,11].

As with all disparities in the field of health protection, inequities in COVID-19 vaccination violate the principle of the universal right to health and, in general, the principle of social justice [12]. This is even more true in health emergencies such as the most recent pandemic, where the objective of protecting populations has become imperative. These differences have had a major influence on the equity of global health and the overall efficacy of the pandemic response, since they have left large portions of the world’s population unprotected from the health and societal repercussions of SARS-CoV-2, allowing its effects to persist unchecked [10].

Aimed at equitable access to COVID-19 vaccines, an initial global initiative for dose-sharing was the creation of the COVAX platform, co-led by the WHO, Gavi, the Vaccine Alliance, and the Coalition for Epidemic Preparedness Innovations (CEPI). As one of the pillars of the Access to COVID-19 Tools (ACT) Accelerator, COVAX was established as a partnership model of cooperation between public and private entities to ensure access to doses [11]. Later, in January 2022, WHO, UNICEF, and Gavi established the COVID-19 Vaccine Delivery Partnership (CoVDP) to provide urgent operational support to those countries that had full vaccination coverage at or below 10% [13].

Despite these efforts, obstacles endure, and the attainment of just distribution of COVID-19 vaccinations continues to be elusive [9]. According to data from the World Health Organization (WHO), the percentage of the total population vaccinated with at least one dose of a COVID-19 vaccine varies significantly among different countries worldwide. By the end of 2023, only 10 countries had achieved a population coverage exceeding 90%. The highest percentages were recorded in Western Europe and the Americas, albeit with some exceptions. Further down the list, it is LMICs, especially from Africa, that populate the group with the lowest percentages of the population having received at least one dose of a COVID-19 vaccine [1]. Furthermore, although on 5 May 2023, the WHO officially declared the end of the COVID-19 emergency, the ongoing viral circulation and the emergence of new variants mean that the administration of booster vaccinations has become a critical component of public health strategies to maintain immunity and control the spread of the virus [14]. In the context of COVID-19 vaccine inequalities, another issue that is clinically and epidemiologically relevant is the increase in the number of SARS-CoV-2 variants resulting from insufficient vaccination coverage, in terms of the properties of the variants’ transitions (e.g., speed, timing, and magnitude of the transition) [15]. Therefore, addressing vaccination inequalities is crucial to controlling the spread of the virus and preventing the development of further variants that can undermine global vaccination efforts. Again, the emergence of new variants has also prompted updates in booster vaccine formulations, emphasizing the need for continuous monitoring and adaptation of vaccination strategies, especially targeted at vulnerable populations [16]. Also in this case, trends indicate that booster uptake varies significantly across different regions and demographic groups, often reflecting broader disparities in access to healthcare and the vaccines’ distribution that have pervaded all phases of the pandemic, with LMICs, rural areas, and equity-deserving groups struggling with lower booster uptake due to limited vaccine supplies, logistical challenges, and ongoing vaccine hesitancy [17,18].

Considering the above, understanding and mitigating the factors that drive these disparities is paramount for public health strategies aiming to ensure equitable distribution and uptake of the vaccines. The goal of this review was to examine these multifaceted problems, providing insights into the factors that impede and facilitate vaccine equity, as well as offering guidance on how to promote more equitable and successful vaccination programs.

## 2. Determinants of Equity in Vaccination

Equitable access to vaccines is influenced by a wide array of factors operating at global, regional, national, and individual levels, which influence the complex landscape of the vaccine’s distribution, accessibility, and acceptance among populations and groups. Indeed, understanding and addressing these determinants is key for devising strategies that promote fairness in the vaccine’s distribution, particularly in the context of the COVID-19 pandemic [7,19,20]. Each determinant can contribute to various aspects of vaccine equity, creating a multifaceted web of challenges that need to be investigated. The individual analysis of each of these determinants is certainly one of the most complex aspects when discussing vaccines (not just the COVID-19 vaccine) in public health. These determinants vary across contexts, historical periods, social groups, and types of vaccine offerings. Moreover, they rarely act in isolation, often working with the complexity and interdependence of these factors to determine who receives the appropriate immunological protection against preventable diseases and when.

With this premise, this focus aimed to briefly examine the most relevant determinants that have operated during—and continue to influence—the COVID-19 vaccination campaign, with the goal of providing a broad overview of the key points related to this topic.

Primary determinants are linked to socioeconomic factors, whose health impact is well-documented in literature across a wide range of health indicators. People who are disadvantaged in terms of income, educational attainment, and employment have poorer access to healthcare resources, including vaccines [12].

Differences in the uptake of COVID-19 vaccination emerged by income, even when the costs of vaccination were entirely covered by the state. This is because low income serves as a proxy for other factors contributing to health inequality. Studies have suggested that even with vaccination being free of charge, the association with low-paying or unstable jobs could lead people to prioritize immediate financial needs over healthcare, face inability to take time off work, and avoid the indirect cost of traveling to a clinic to receive the vaccine doses, leading to lower vaccination rates. Furthermore, individuals with low income, lacking insurance, were less likely to receive advice from a primary care provider about the necessity of taking COVID-19 vaccines and boosters [21,22,23,24,25,26]. Furthermore, individuals with lower income are more likely to be forced to live in underserved areas that typically have fewer healthcare infrastructures, including COVID-19 vaccination sites, than urban areas [27,28]. This is particularly significant, because they also tend to live in crowded or substandard housing, thereby facing higher exposure to infectious diseases transmitted through direct contact, such as COVID-19 [29].

Education has also been associated with vaccine uptake [30]. The heterogeneity of COVID-19 vaccination-related behaviors has shown that higher educational levels are linked to better understanding and acceptance of vaccines, as an indirect effect of an improved ability to navigate the healthcare system effectively. Conversely, lower educational attainment and health literacy affects individuals’ ability to understand health information and can lead to a lack of adequate information regarding the benefits and schedules of vaccination, and, therefore, to vaccine hesitancy [31,32,33].

Another significant determinant of access to and receipt of the COVID-19 vaccination, and intention to vaccinate is ethnic identity. Extensive research has consistently shown that minority groups, particularly those with distinct national or cultural traditions differing from the majority population of a country, experience lower vaccine uptake and completion rates [28,34,35,36,37]. This discrepancy in actual vaccine uptake among these groups directly correlates with levels of vaccine hesitancy and resistance, which are often exacerbated by socioeconomic disadvantages that marginalized communities frequently encounter. Among these are low levels of educational attainment and income, precarious working conditions, inadequate social support, systemic barriers to access to healthcare, including language barriers, discrimination, and a pervasive lack of trust in medical institutions. These barriers not only impede their access to vaccination but also amplify their vulnerability within the healthcare system [37,38,39,40]. Furthermore, members of racial and ethnic minority groups face disproportionately higher risks of COVID-19 positivity and disease severity, linking directly to the same social and economic determinants. Studies have highlighted a stark gradient in how these factors influence the outcomes of COVID-19, underscoring that the disparities extend beyond access to and attitudes towards vaccination [41].

Social networks and community influence, political ideology, and affiliation with certain religious groups also appear to be involved in shaping judgments of COVID-19 vaccines’ benefits, utilities, and risks. These factors, either individually or in association with each other and other social determinants of health, have been linked to the consumption of health information from informal sources, mainly social media reports and peers. These sources are likely to be biased by a high content of false and partial news about the risks of COVID-19 and the vaccine, and moderate the belief of being falsely protected, since the individual’s health is seen as dependent on external factors other than healthcare providers and vaccination programs [42,43,44,45,46,47,48,49,50].

Health infrastructure and public factors play crucial roles in shaping vaccine equity too. Well-funded and well-organized health systems ensured the widespread and equitable distribution of COVID-19 vaccines. In contrast, under-resourced systems struggled to reach all segments of the population [51]. This disparity was further exacerbated by logistical challenges, such as cold chain requirements and healthcare facilities’ limitations, which hindered the vaccines’ rollout in resource-constrained settings [52].

In the case of COVID-19 vaccines, it is also difficult to exclude an effect of the type of vaccine, among those developed since 2020, on the observed differences in vaccinations, although further research is needed on this point. However, some aspects should be considered regarding how the type of vaccine available could directly or indirectly exacerbate or mitigate the existing inequities. High-income countries (i.e., those with a robust cold chain infrastructure) have better access to a variety of vaccines, including mRNA platforms, while LMICs may rely on vaccines that are easier to distribute but potentially less effective, thereby impacting the overall public health response [53,54,55]. Similarly, the different perceived efficacy of a type of vaccine can affect public willingness to be vaccinated [56].

Given the complexity of each of these determinants of equity in vaccination and their synergistic interplay, a thorough analysis requires resources and interdisciplinary collaboration. Addressing each determinant in isolation is, however, insufficient; instead, a holistic approach that considers their interplay is vital to developing comprehensive and inclusive strategies. This approach ensures that vaccination efforts, especially for COVID-19, are equitable and effective in protecting all populations, regardless of their circumstances, and that everyone has fair access to life-saving vaccines. A relevant point is the fight against vaccine hesitancy. It is a complex phenomenon and tends to be context-specific. Its contribution to COVID-19 vaccine disparities lies in the synergistic interaction of the socioeconomic and cultural inequalities mentioned in this paragraph; specifically, misinformation, distrust in the government and healthcare systems, and historical injustices, particularly among marginalized groups and those with lower educational attainment exacerbate hesitancy. To address the underlying factors, targeted public health interventions, including transparent communication, engagement with the community, and culturally sensitive education campaigns, are required to build trust and promote acceptance of the vaccine across diverse populations [57].

## 3. Disparities in COVID-19 Vaccination

The global COVID-19 vaccination campaign has starkly highlighted regional and country-level disparities in coverage rates, underscoring significant inequalities in public health infrastructure and socioeconomic conditions. These disparities are primarily driven by variations in vaccine supply, the maturity of healthcare infrastructure, and the level of political commitment across different regions [58].

As mentioned earlier, LMICs have faced considerable vaccine shortages, largely because acquisition of the vaccine relied heavily on donations and purchases through COVAX—a global vaccine-sharing mechanism aimed to ensure equitable access to vaccines for all countries regardless of their income level [1,59]—which, however, have been very competitive [60,61]. In stark contrast, high-income countries were able to secure large quantities of vaccines early on through advance purchase agreements and robust healthcare systems, leading to higher vaccination rates and faster immunization rollouts [9]. As a direct consequence, a data-driven, age-stratified epidemic model assessed the effects of vaccine inequity in 20 LMICs, suggesting that over 50% of deaths could have been prevented with higher or earlier availability of the vaccine [62].

While it would be beneficial to have a numerical quantification of the problem, the difficulties in obtaining trustable data, reaching all equity-deserving groups and addressing problematic contexts make this challenging. The lack of reliable and comprehensive data, particularly from underserved and marginalized populations, hinders accurate measurement and analysis. To obtain an overview of the problem, a useful source is the publication by Gozzi et al., in which the authors developed a stochastic, multi-strain compartmental epidemic model applied to 20 LMICs worldwide to quantify access to and administration of the vaccine across different income levels. The authors estimated that, as of 1 October 2022, 77% of individuals in high- and upper-middle-income countries had completed their initial COVID-19 vaccination course, compared with 50% in LMICs. This disparity was even more pronounced during the early stages of the vaccines’ rollout. The analysis also showed a strong positive correlation between a country’s Human Development Index and its vaccination coverage. Furthermore, mathematical models suggest that had LMICs achieved vaccination rates equivalent to those of high-income countries, a significant percentage of COVID-19 deaths could have been averted. Counterfactual scenarios also indicate that earlier initiation of vaccination and stricter non-pharmaceutical interventions could have significantly reduced death rates in these countries [62].

The complexity of procurement of the COVID-19 vaccine was the first layer contributing to regional disparities in vaccination. Indeed, the procurement system played a critical role in shaping regional disparities in the coverage of vaccination, influencing not only the availability of vaccines but also the efficiency and equity of their distribution [63]. This system encompasses the various mechanisms and processes involved in acquiring vaccines, from decision-making and budgeting to the storage and final delivery of the vaccines to the end-users. To understand the ways in which these mechanisms affect regional disparities, a rapid exploration of the procurement cycle and its related obstacles is necessary. Procurement may be centralized, where a supra-national entity negotiates and purchases vaccines on behalf of all nations within a geographical area, or decentralized, where regions, local entities, or individual nations manage their own procurement. Centralized procurement often benefits from economies of scale, potentially leading to lower per-unit costs and more standardized delivery processes. However, it may also result in uneven distribution if supra-national priorities overlook national needs or if logistical planning is not attuned to each specific context. Decentralized procurement allows regions to tailor their vaccine orders to reflect their local health priorities and demographic needs more closely. However, this method may suffer from a lack of bargaining power and higher prices per dose, which can be particularly disadvantageous for economically weaker regions [63,64,65,66].

Disparities in COVID-19 vaccination are evident not only on an international scale but also within countries, and are particularly pronounced between rural and urban areas, especially in LMICs. These disparities have contributed to the varying distribution of vaccination coverage across different socioeconomic levels worldwide [51]. Urban areas typically benefit from better transportation and healthcare infrastructure, more vaccination sites, and greater public awareness campaigns, all of which facilitate higher vaccination uptake. Conversely, rural areas often face logistical challenges, including such as poor transportation networks and inadequate vaccine storage facilities, lower accessibility, and higher levels of vaccine hesitancy, often fueled by misinformation and lower health literacy [67,68,69]. This is especially critical for COVID-19 vaccines, which require cold chain management; delays in transport can compromise the vaccines’ efficacy if proper temperature controls are not maintained. While urban healthcare facilities usually have modern equipment and reliable electricity supplies to ensure proper storage of the vaccine [52], rural facilities often struggle with frequent power outages and a lack of advanced refrigeration equipment, leading to increased risks of vaccine spoilage [70]. This leads to increased risks of vaccine spoilage, further hindering the ability to conduct sustained vaccination campaigns in these areas. Moreover, the overall healthcare infrastructure in rural areas often lacks the robustness of urban systems. Many rural healthcare facilities are understaffed and underfunded, lacking medical professionals trained to administer vaccines and handle potential adverse reactions [71]. The sparse distribution of healthcare facilities also means that rural residents may have to travel long distances to be vaccinated, posing a significant barrier to achieving high vaccination coverage [72]. These challenges are further compounded by economic constraints that limit the ability of rural governments to invest in necessary the improvements in the infrastructure [73]. Addressing these multifaceted issues requires a comprehensive approach to ensure equitable distribution of and access to vaccines across all regions.

## 4. The Global Fight against Inequalities in COVID-19 Vaccination

Throughout the COVID-19 pandemic, socioeconomic disparities have been linked to a higher and disproportionate burden of COVID-19, and addressing structural inequities in the distribution of the COVID-19 vaccine has been crucial in mitigating the pandemic’s impact [74,75]. Addressing global vaccination disparities requires international cooperation and solidarity. Global initiatives for sharing vaccines have been implemented with the aim of guaranteeing fair access to vaccines across all countries, irrespective of their economic status.

The first of these initiatives was COVAX, spearheaded by the WHO, Gavi (the Vaccine Alliance), and the CEPI, which was dedicated to promoting equitable access to COVID-19 vaccines worldwide. The initiative’s objective is to ensure that every nation, regardless of its financial resources, has equitable access to COVID-19 vaccines. COVAX operates by pooling resources to procure vaccines and distribute them to participating countries, prioritizing those most at risk and those from low- and middle-income economies. The initiative aims to counteract vaccine nationalism by ensuring that vaccines are distributed according to public health priorities rather than financial or political considerations. By striving for global immunity, COVAX seeks to enhance efforts to control the pandemic on a global scale [76]. The mechanism was designed to allocate the available doses to countries in proportion to their total population size, ensuring a steady pace. High-income countries participate through a self-financing mechanism, while LMICs receive doses through donations facilitated by the Advanced Market Commitment (AMC) investment plan. The initiative aimed to vaccinate up to 20% of the population in each participating country [77]. It allowed high-income countries to procure doses for up to 50% of their population through the Optional Purchase Agreement, while for assisted countries, the limit was set at 20%. However, this approach has raised concerns about equity, a principle central to COVAX’s mission [78].

In early 2022, despite 12 billion doses of COVID-19 vaccines being shipped worldwide, only 13% of the population in low-income countries and 8% in countries facing humanitarian emergencies were fully vaccinated. Recognizing the urgency of converting vaccine doses into protected communities, WHO, UNICEF, Gavi, and the Vaccine Alliance launched the COVID-19 Vaccine Delivery Partnership (CoVDP) in January 2022. The initiative aims to support in-country delivery of the vaccine in 92 countries eligible for the AMC, with a focus on the 34 countries experiencing the lowest rates of vaccination coverage [79]. CoVDP focused primarily on facilitating the final stages of the process of distributing the vaccine and conducted a top-level mission to Cameroon to evaluate progress and advocate for measures to resolve bottlenecks [80].

While COVAX serves as the vaccine arm of the Access to COVID-19 Tools (ACT) Accelerator, launched in April 2020, its primary goal is to accelerate the development, manufacture, procurement, and equitable distribution of COVID-19 vaccines worldwide. CoVDP focuses specifically on the final stage of the vaccine distribution chain: ensuring that vaccines are effectively delivered within countries. This initiative prioritizes country engagement, demand forecasting, funding for the logistics of delivery, coordinating distribution efforts, and ongoing monitoring. CoVDP collaborates closely with the partners of COVAX, its mandate extends beyond COVAX-procured vaccines, encompassing vaccines secured through the Africa Vaccine Acquisition Task Team, other partners, or bilateral agreements [13,79].

In addition to procuring and distributing vaccines, COVAX, CoVDP, and other initiatives emphasize the importance of addressing logistical challenges, ensuring fair access to vaccines, and overcoming obstacles such as vaccine hesitancy and misinformation. International cooperation and solidarity are essential to tackle global vaccination inequalities. Initiatives such as COVAX aim to guarantee equitable access to vaccines for all countries, regardless of their income level. Despite these challenges, the allocation of COVID-19 vaccines should prioritize justice by ensuring equitable access at national and regional levels. Policymakers must commit to removing unjust and avoidable barriers to vaccination, particularly focusing on reducing health inequities among populations experiencing higher COVID-19 morbidity and mortality [81].

Vaccine procurement involves a complex system that necessitates ongoing collaboration among public health professionals, national policymakers, international regulators, and manufacturers to tailor procurement strategies to local needs [63]. However, bridging these gaps necessitates a coordinated effort among international organizations, national governments, and the private sector. A critical area of focus is enhancing mechanisms of vaccine procurement to prevent shortages and ensure equitable access. This involves not only securing reliable vaccine supplies through fair contracts but also enhancing the healthcare infrastructure and improving logistics and distribution networks to reach even the most remote areas [82,83]. Additionally, facilitating the transfer of technology and implementing intellectual property waivers are essential steps to boost the capabilities of vaccine production in LMICs. These measures can significantly increase the global vaccine supply and help minimize disparities, ensuring that vaccination efforts are inclusive and effective across all regions [84,85].

## 5. Equity in Vaccination at National and Local Levels

### 5.1. Lessons Learnt at National and Local Levels to Reduce Inequalities

Vaccine equity is, by definition, fair and equitable access to vaccines for all people regardless of the social group to which they belong or factors such as racism, homophobia, social class, or sexism [86]. Well-coordinated national and local strategies to promote equity in access to vaccines are essential to ensure that all people, regardless of their socioeconomic or demographic situation, have the opportunity to be immunized against COVID-19 [87].

During the global vaccination campaign, different countries adopted various strategies to address the determinants of uptake of the COVID-19 vaccine, aiming to improve vaccination rates and close health gaps among different populations and groups. The success of these strategies has varied, with some examples standing out more notably than others. For instance, Rwanda emerged as a success story by efficiently utilizing the opportunities provided by COVAX, making the country an example of effective pandemic control and achieving a successful rollout of the COVID-19 vaccine in the first quarter of 2021 [88]. This success was also due to recent advancements in Rwanda’s vaccination efforts, rooted in initiatives that engaged community health workers and strengthened the health information system. The country’s proactive approach, including rigorous public awareness campaigns and transparent communication, significantly boosted the vaccine’s acceptance and coverage [88,89].

One example of prioritizing marginalized communities in high-income countries is Canada, which effectively reduced disparities through targeted outreach and mobile vaccination units. The initiatives focused on a variety of equity-deserving groups (ethno-racial minorities, people of different faiths, immigrants and refugees, and other vulnerable populations) and were essentially based on the following aspects: (i) to “consult and involve,” where engagement with high-risk groups was achieved through vaccine task forces and working groups that facilitated community engagement and informed certain populations; and (ii) to “collaborate and empower” communities, through community ambassadors and mobilizers who implemented on-the-ground engagement on the vaccine’s availability, safety, and efficacy, directed to equity-deserving groups [90]. Another similar model is that of the Mobile Health Clinics (MHC), developed in the US using a bottom-up engagement strategy with community leaders, which also offered screening and social assistance campaigns to the most disadvantaged populations (i.e., “hard-to-reach” groups). This model led to an increase in coverage among various groups, especially in the Latino population [91].

Regarding the capillarity of the offer of vaccination and the proximity of vaccination sites, a systematic review by Romero-Mancilla et al. found that vaccinations through pharmacies promote the accessibility of vaccines and territorial equity throughout the world, in addition to the fact that, generally, a relationship of trust is established with the pharmacist, which increases vaccination compliance [92].

Several community interventions to reach minority groups through initiatives aimed at combating misinformation and increasing trust and access to COVID-19 vaccination came from the US. For instance, a rapid review by Dada et al. provided a basis for developing strategies that promote equity in COVID-19 vaccine uptake for Black communities [86]. In Florida, a pastor at the Baptist Church of Tallahassee convened a statewide COVID-19 vaccine community outreach task force that included historically Black representatives from colleges and universities, business leaders, media representatives, and politicians. They partnered with the state government to provide vaccinations to Tallahassee’s Black communities and underserved neighborhoods. In Detroit, a city where more than three-quarters of all residents are Black and vaccination rates were five percentage points lower than the rest of the state as of January 2021, they solved the problem of transportation by hiring private companies to provide free transportation to vaccination sites, opened more vaccination appointments, and hired additional providers [86].

Again with regard to minorities, in some tribes of the US, such as American Indians and Alaskan Natives, financial incentives have been used to increase vaccination rates [93], while the Australian government has supported a free vaccination strategy and has also expanded the availability of vaccine suppliers to include Aboriginal and Torres Strait Islander pharmacists and health workers to increase adherence to vaccination in these populations [94].

On the other hand, some countries faced challenges that highlighted critical areas for improvement. Amongst others, in India, during the various phases of the rollout, the vaccine’s distribution was hampered by logistical issues and supply shortages, particularly in rural regions, underscoring the need for better supply chain management [95]. In Brazil, which struggled with political turmoil, the message about COVID-19 vaccinations was widely politicized, with inconsistent—or, worse, denigratory—public messaging that led to vaccine hesitancy and uneven coverage. This highlighted the importance of cohesive policy and trust-building in public health initiatives; most importantly, it provided elements for studying the pervasive association between political ideology and disparities in COVID-19 vaccination [96].

Finally, vaccination equity between socially, culturally, and economically different populations can be achieved; however, great effort and targeted community commitment are needed, including initiatives aimed at ensuring vaccination services that are suitable for the population and place, vaccination incentive campaigns on social media and through institutional sites, and the involvement of opinion leaders, all tailored to the groups being targeted.

### 5.2. National and Local Strategies to Promote Equity in Vaccination

These experiences underscore that while a strong infrastructure, targeted outreach, and clear communication are crucial for reducing vaccine inequalities, overcoming logistical and political barriers remains a significant challenge. They also serve as reference to outline possible solutions. At the national level, governments can implement policies that prioritize distribution of the vaccine to vulnerable and underserved communities, including low-income groups, ethnic minorities, and rural populations. This involves creating equitable allocation frameworks, enhancing transparency in the distribution of the vaccine processes, and establishing mobile vaccination units to reach remote areas. Additionally, national public health campaigns can be launched to combat misinformation and build trust in vaccines, utilizing diverse media platforms and culturally tailored messaging. Defining all possible strategies in detail is challenging due to the multifaceted nature of these interventions; however, we can categorize them into six macro areas as follows.

i. Planning and equitable allocation of vaccine supplies: National governments must establish guidelines for allocation of the vaccine that consider the specific needs of different regions and populations. This may include distribution based on population density and the availability of health resources [97,98].
ii. Accessibility of vaccination sites: It is essential that vaccination sites are easily accessible to all people, especially those living in remote or disadvantaged areas. National governments can work with local authorities to establish vaccination sites in the most vulnerable communities and provide free transportation for those who need it [28,99].
ii. Targeted programs for marginalized populations: National governments should implement targeted programs to reach marginalized, fragile, or at-risk populations. These programs may include distributing vaccines at community centers, churches, schools, or other locations frequented by the target population, as well as providing information and language support to overcome cultural and linguistic barriers [100,101,102,103].
iv. Monitoring and evaluation of vaccination programs: National governments should constantly monitor the implementation of vaccination programs and evaluate equity in access to the vaccine. This can be achieved through analyzing demographic data and collaborating with local and community organizations to identify and address emerging disparities [104].
v. Clear and accessible information: Information about vaccinations must always be clear, accurate, and accessible, including details on the benefits, risks, and distribution process of the vaccines. Mitigating misinformation and increasing public trust in the safety and effectiveness of vaccines also increases adherence to vaccination campaigns [105].
vi. International collaboration: Collaboration between nations and international organizations helps ensure equitable access to vaccines globally. This may include participation in initiatives such as the COVAX Facility, which aims to ensure equitable access to vaccines worldwide, as well as the exchange of improved procedures that can be applied in different countries [13].

Moreover, local governance of the distribution of vaccines has a crucial impact on equity in vaccines, as it relies on a series of public and private actors working together synergistically [106,107]. Local strategies to promote equity in access to vaccines are essential to ensuring that all communities, especially marginalized or disadvantaged ones, can be adequately immunized against COVID-19 [108]. By combining top-down policy initiatives with grassroots community efforts, it is possible to create a comprehensive approach that promotes equity in COVID-19 vaccination and enhances public health outcomes. Local authorities should address the following.

i. Targeting priority population groups: Local authorities should work with community organizations and interest groups to identify at-risk populations within their area. This may include older people, people with disabilities, low-income individuals, minority ethnic communities, and other disadvantaged groups. Effective methods include the use of detailed demographic and epidemiological data to identify at-risk groups [86].
ii. Establish accessible vaccination sites: Local authorities should establish easily accessible vaccination sites in the most vulnerable communities. These sites should be located in places that are well connected and easily reachable by local residents. Furthermore, they should be adapted to ensure access for people with disabilities and other special needs [109]. Mobile vaccination units and pop-up clinics can increase access in underserved areas [110].
iii. Conduct awareness and information campaigns: It is crucial to conduct awareness and information campaigns to educate communities on the importance of vaccination and to combat misinformation, including among healthcare workers. These campaigns should be linguistically and culturally appropriate, and involve community leaders and trusted figures, ensuring that information on vaccines is accessible and relevant [111,112].
iv. Collaborate with community organizations: Local authorities should work closely with community organizations and voluntary groups to reach the most vulnerable populations. These organizations can play a key role in identifying and referring people who may have difficulty accessing vaccination services [113,114]. Moreover, involving local leaders and organizations helps build trust and ensures culturally sensitive communication [115].
v. Make the best use of technologies to reach target populations: Utilizing digital tools, such as GIS mapping and data analytics, can optimize the logistics of distributing the vaccine and identify gaps in coverage [116].
vi. Ensure the equitable distribution of resources: It is important to ensure that vaccination resources are distributed equitably among different communities and that there are no disparities in access to vaccination services. This may require mobilizing additional resources to address the specific needs of disadvantaged communities [117].
vii. Monitor and evaluate: Local authorities should constantly monitor the implementation of vaccination strategies and evaluate equity in access to vaccines. This can be achieved through analyzing demographic data and gathering feedback from the community [104].

## 6. Conclusions

Equitable access to COVID-19 vaccines is influenced by a myriad of factors operating at global, regional, and national levels. Socioeconomic status, the healthcare infrastructure, political stability, and cultural beliefs all play significant roles in shaping disparities in vaccination. Understanding these determinants is essential for developing effective strategies to promote equity in the distribution of vaccines, which requires collaborative efforts at the local, national, and global levels [118,119].

Achieving equitable access to COVID-19 vaccines is essential for controlling the pandemic and protecting public health. Addressing global, regional, and within-country inequalities in vaccination requires coordinated efforts at multiple levels. By prioritizing equity in the distribution of vaccines and implementing targeted strategies to reach underserved populations, we can work towards a world where everyone has access to life-saving vaccines, regardless of their socioeconomic status or geographic location. The pandemic has underscored the importance of investing in global health infrastructure and preparedness. Strengthening health systems, enhancing surveillance and response capabilities, and fostering international collaboration are vital for addressing not only COVID-19 but also future infectious disease threats [120].

Achieving high vaccination coverage worldwide is essential for preventing further waves of infection and ensuring long-term control of COVID-19 [121]. Similar to all other anti-COVID interventions, vaccination has had and continues to have a profound impact on universal health coverage. It does so through the tools that have accelerated the development and distribution of COVID-19 vaccines, leading to significant advancements in the global health infrastructure, such as the enhancement of cold chain logistics and delivery mechanisms of healthcare. These improvements can be used in the future to accelerate the pace of immunization against other diseases, particularly in contexts and among populations where this health need is still unmet. At the same time, within the scope of universal health coverage, the inequitable distribution of vaccines has highlighted and, in some cases, worsened global health inequalities, undermining efforts to achieve universal health coverage. To fully capitalize on the positive impacts and mitigate the negative ones, it is crucial to integrate COVID-19 interventions into comprehensive health strategies that prioritize equity and resilience in health systems [122,123].

In a nutshell, the COVID-19 pandemic has highlighted deep-seated inequities in global health, and equitable access to vaccines is crucial for overcoming these challenges. While relevant progress has been made, much work remains to ensure that all countries can protect their populations and achieve a durable end to the pandemic. Addressing vaccine inequity requires a multifaceted approach including financial support, policy changes, and international cooperation to ensure that no one is left behind in the fight against COVID-19. This review highlights the importance of addressing the socioeconomic determinants, vaccine hesitancy, and logistical barriers to achieve universal vaccination coverage. By learning from country-level experiences and implementing targeted strategies, we can reduce inequalities and improve health outcomes. The integration of COVID-19 vaccination efforts into broader health coverage initiatives is essential for building resilient health systems capable of responding to future pandemics. Policymakers must prioritize equity in the distribution of vaccines and leverage international cooperation to ensure that no population is left behind.
